# Supplementary figures and images for: Bacterial Strain–Dependent Dissociation of Cell Recruitment and Cell-to-Cell Spread in Early M. tuberculosis Infection
Source: mBio. 2022 Jun 13;13(3):e01332-22. doi: 10.1128/mbio.01332-22 (PMC9239178; doi:10.1128/mbio.01332-22)

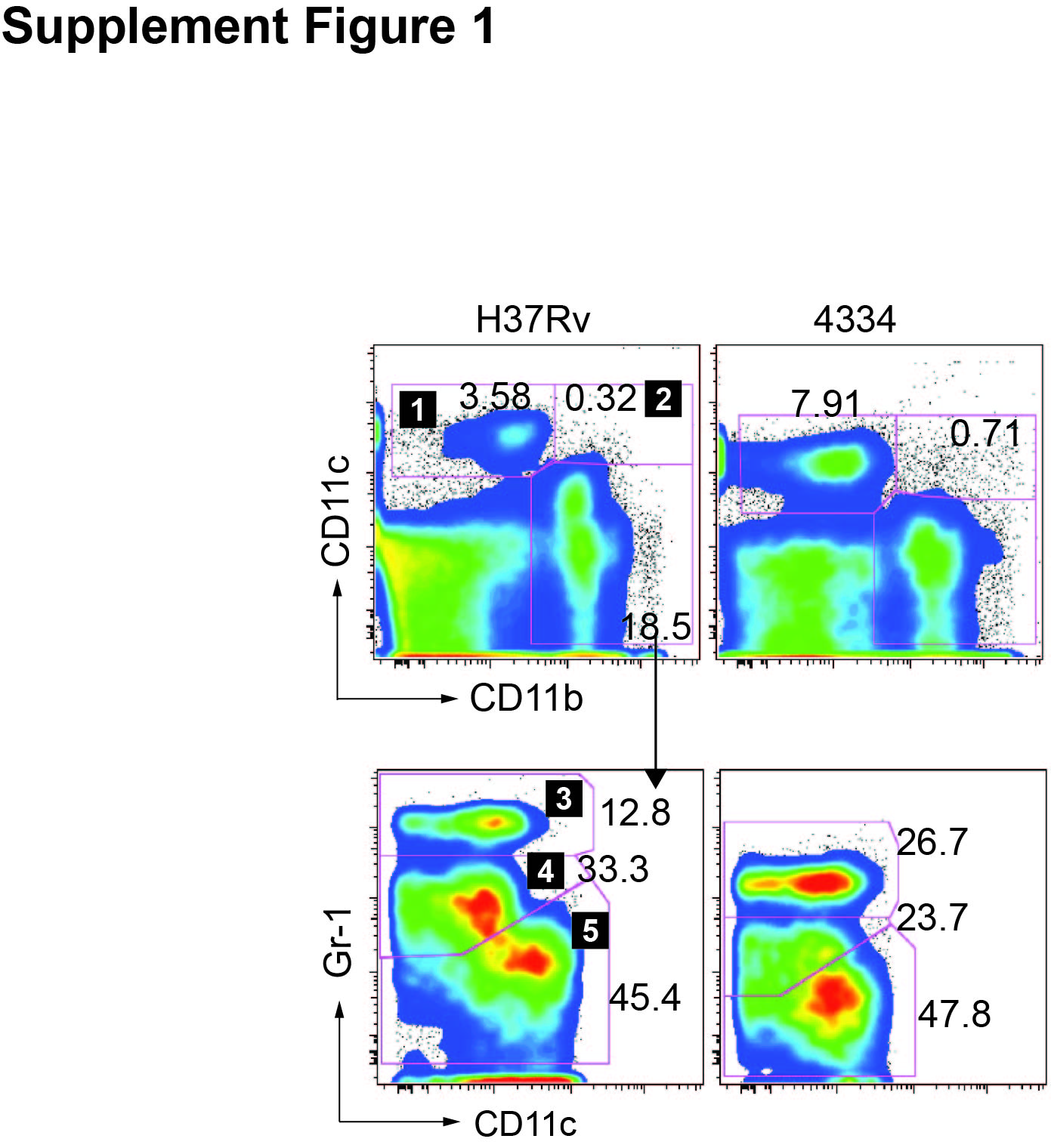

Supplement: FIG S1 [file mbio.01332-22-s0005.jpg]

Supplement Figure 2

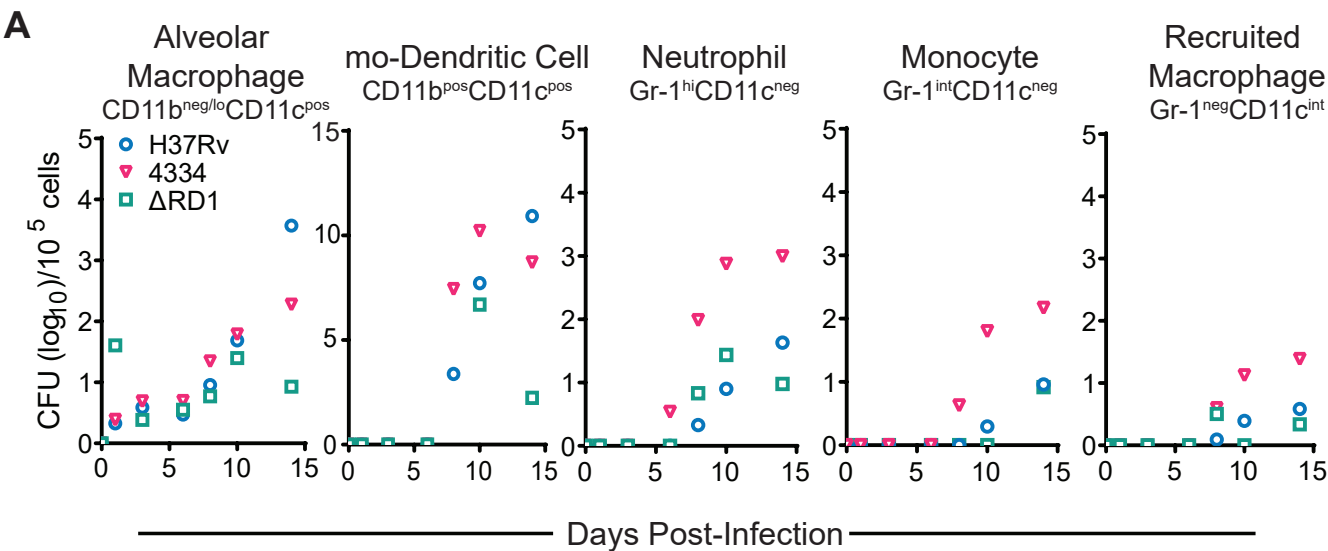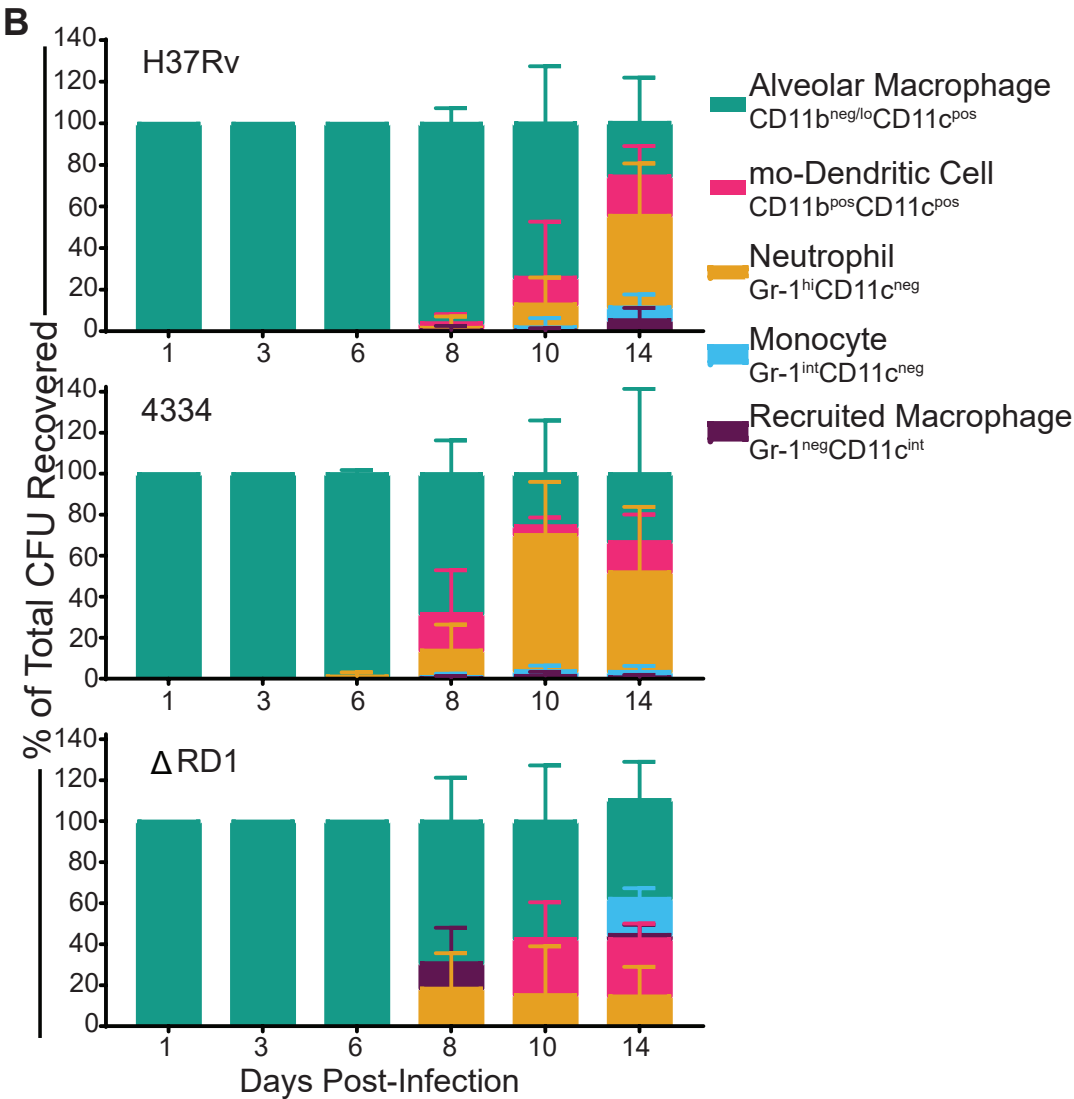

Supplement: FIG S2 [file mbio.01332-22-s0001.pdf]

## Supplement Figure 3

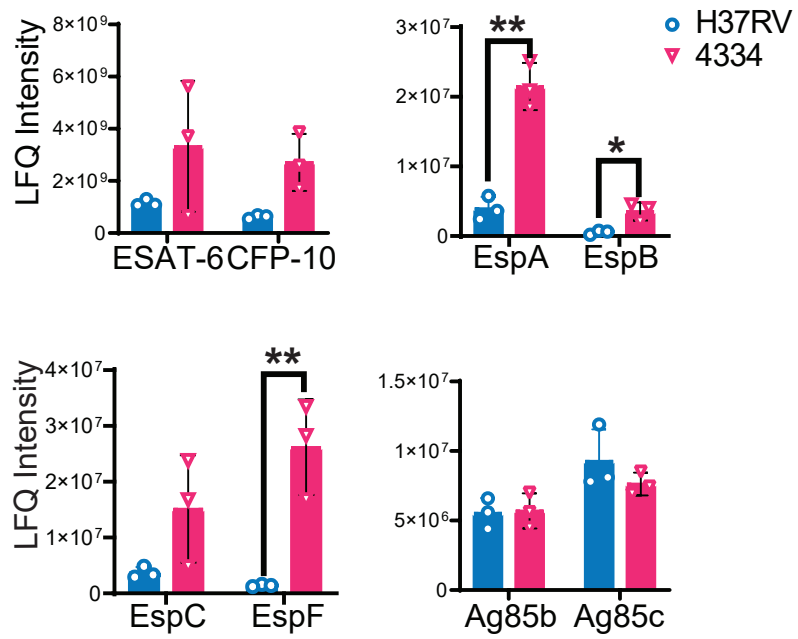

Supplement: FIG S3 [file mbio.01332-22-s0002.pdf]

## Supplement Figure 4

**A**

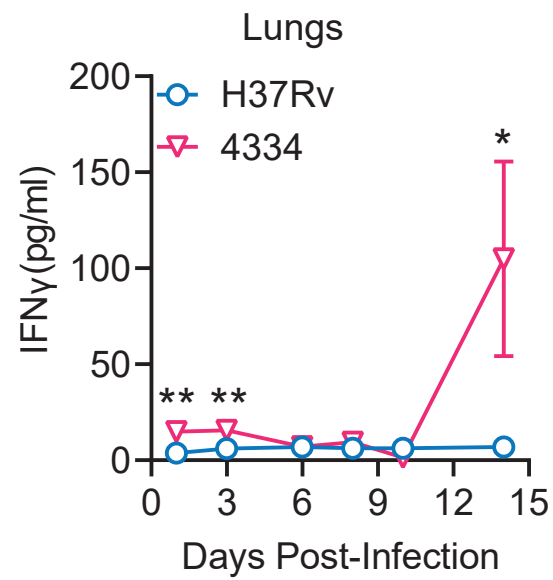

**B**

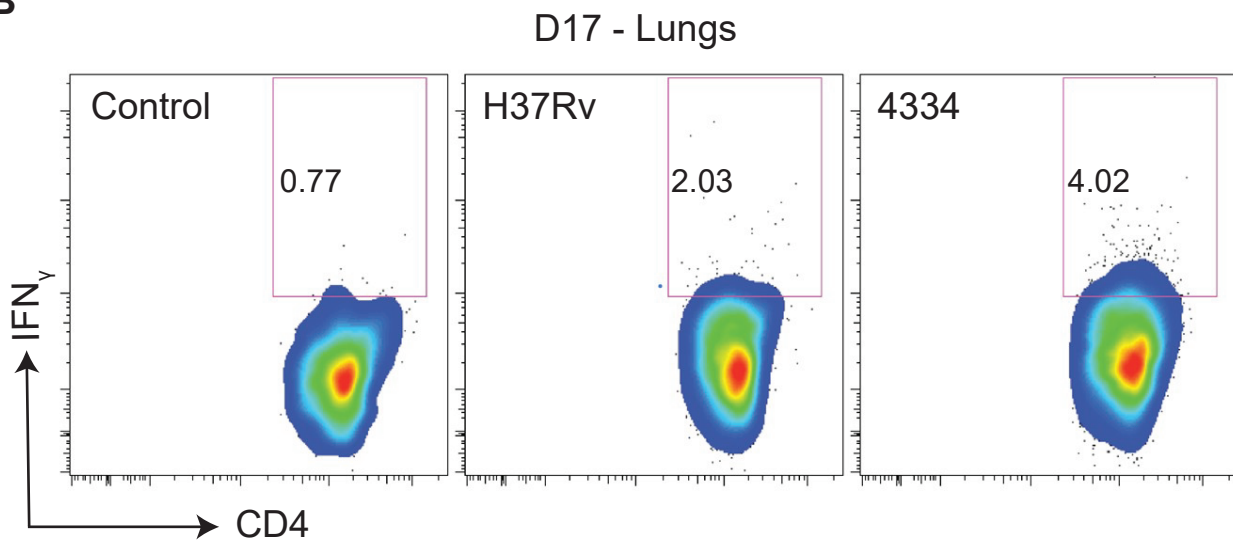

Supplement: FIG S4 [file mbio.01332-22-s0003.pdf]

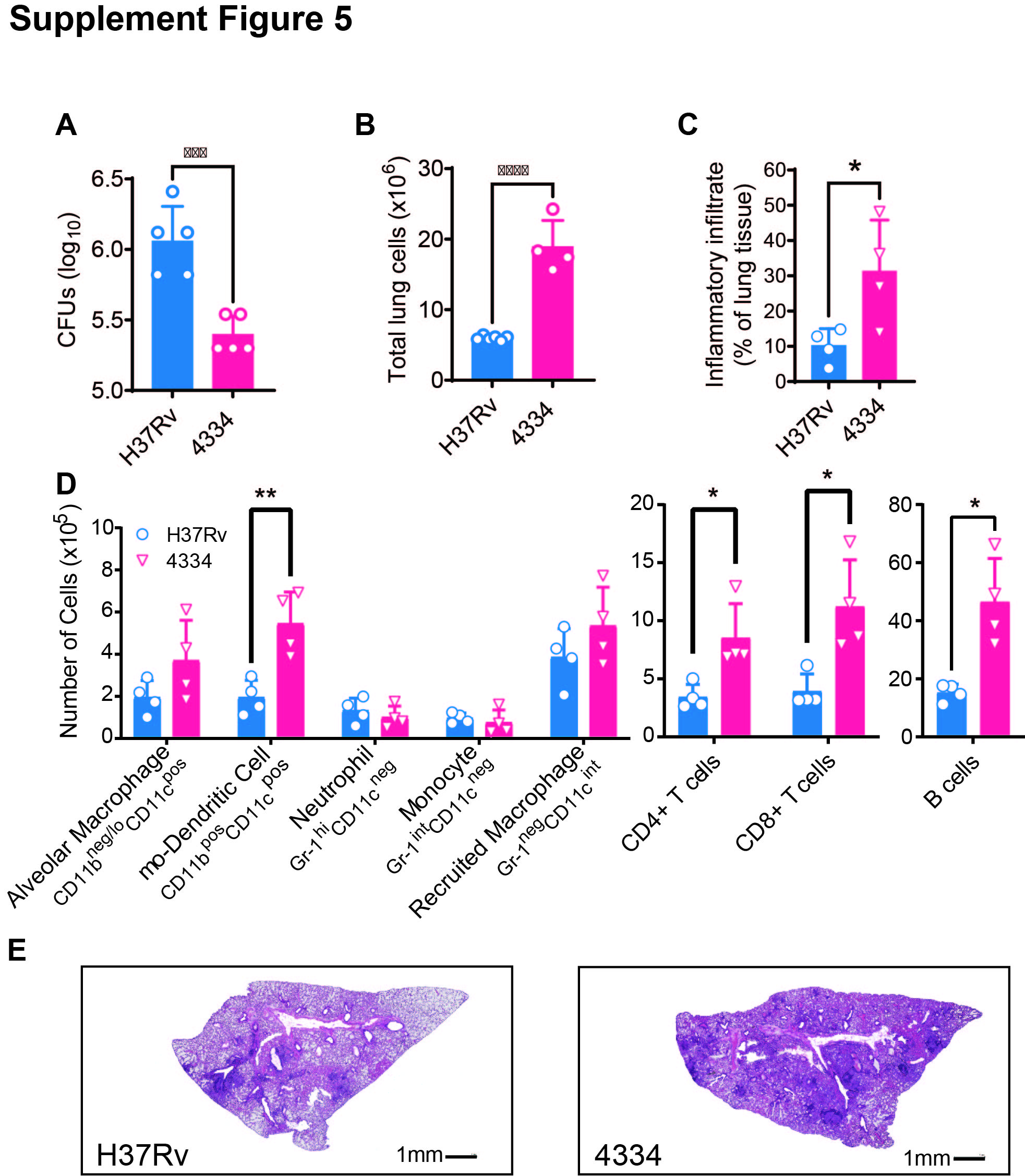

Supplement: FIG S5 [file mbio.01332-22-s0004.jpg]
